# Supplementary material for: Photo-Functionalized Magnetic Nanoparticles as a Nanocarrier of Photodynamic Anticancer Agent for Biomedical Theragnostics
Source: Cancers (Basel). 2020 Mar 1;12(3):571. doi: 10.3390/cancers12030571 (PMC7139909; doi:10.3390/cancers12030571)
Supplement: Supplementary file 1 [file cancers-12-00571-s001.pdf]

cancers

Supplemental Materials: Photo-functionalized magnetic nanoparticles as a nanocarrier of anticancer agent for biomedical theragnostics

Table 1. FCF NP concentration-dependent T2 relaxation rate in each cancer cell.

Concentraiton of FCF (ug/ml)	HeLa	MCF-7	PC-3	SKOV-4
	1/T2 (s^{-1})			
0	2.83	2.05	1.47	3.76
20	5.77	4.18	4.40	5.24
40	7.78	5.34	5.41	6.98
60	8.74	6.71	5.32	8.18
80	11.45	7.94	8.50	10.52
100	15.31	8.61	8.70	10.89
120	15.06	8.90	9.94	12.09
140	19.16	9.65	10.64	14.01
160	19.88	11.36	13.04	15.06
180	20.92	10.20	13.14	17.95
200	21.93	12.29	13.68	18.18
220	28.99	11.81	14.49	20.16

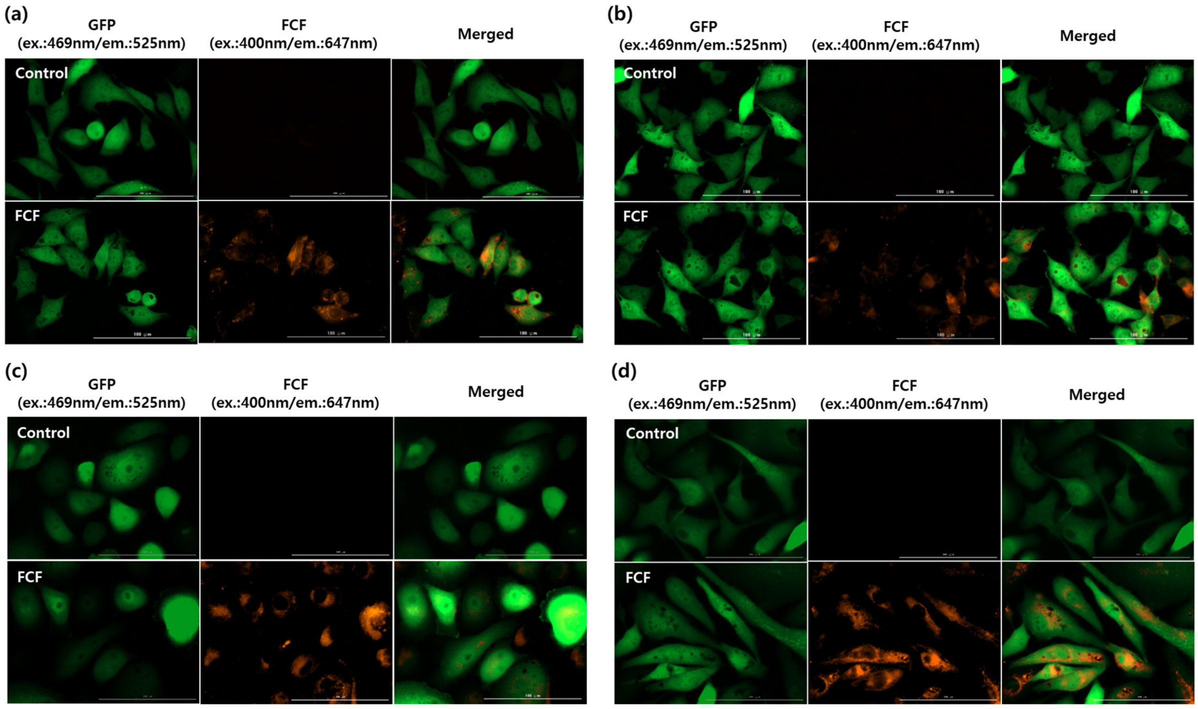

Figure S1. Fluorescence imaging of FCF NP-treated cancer cells. (**a**) HeLa, (**b**) MCF-7, (**c**) PC-3 , and (**d**) SKOV-3 cells. Fluorescence images of green fluorescent protein (GFP)-transfected cancer cells taken after 2 h incubation with 20 μg/mL Fe_3O_4-Ce_6-FA nanoparticles (FCF). The images were taken

using a 40x objective lens and fluorescence optics (excitation/emission at 377/447 nm for GFP and 400/647 nm for FCF). Scale bar = 100 μm.

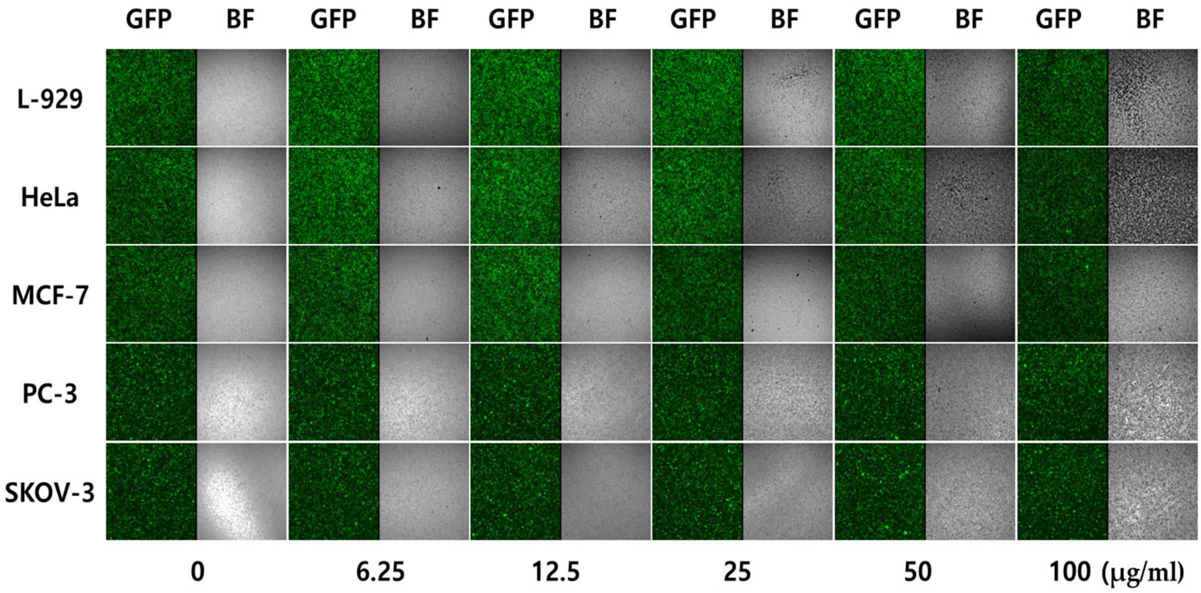

Figure S2. Fluorescence imaging for *in vitro* cytotoxicity of FCF NPs in various cell lines. Fluorescence cellular images were acquired using a 10x objective lens and fluorescence optics (excitation at 377 nm and emission at 447 nm) after incubation for 24 h under dark conditions. Scale bar = 100 μm. GFP: Green fluorescent protein; BF: bright field.

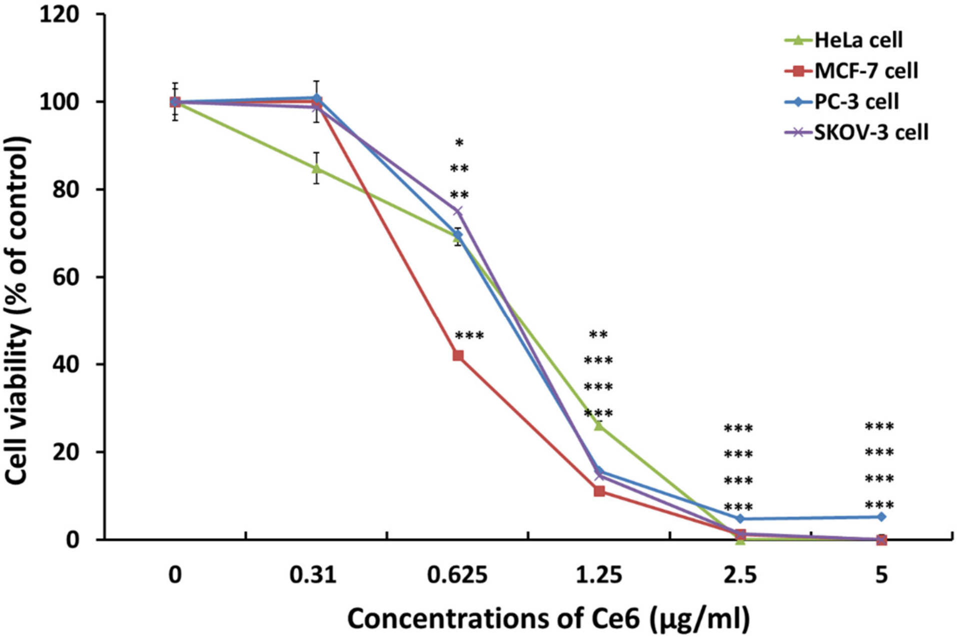

Figure S3. Photodynamic anticancer activity of Ce6 in various cancer cells. Phototoxicity of Ce6 in HeLa, MCF-7, PC-3, and SKOV-3 cells over 10-min exposure to 40 mW LED light. Data for photodynamic anticancer activity are expressed as mean ± S.D. (n = 4). Statistical significance was analyzed by Student's *t*-tests. *$p < 0.05$, **$p < 0.005$, ***$p < 0.0005$ (vs. control).

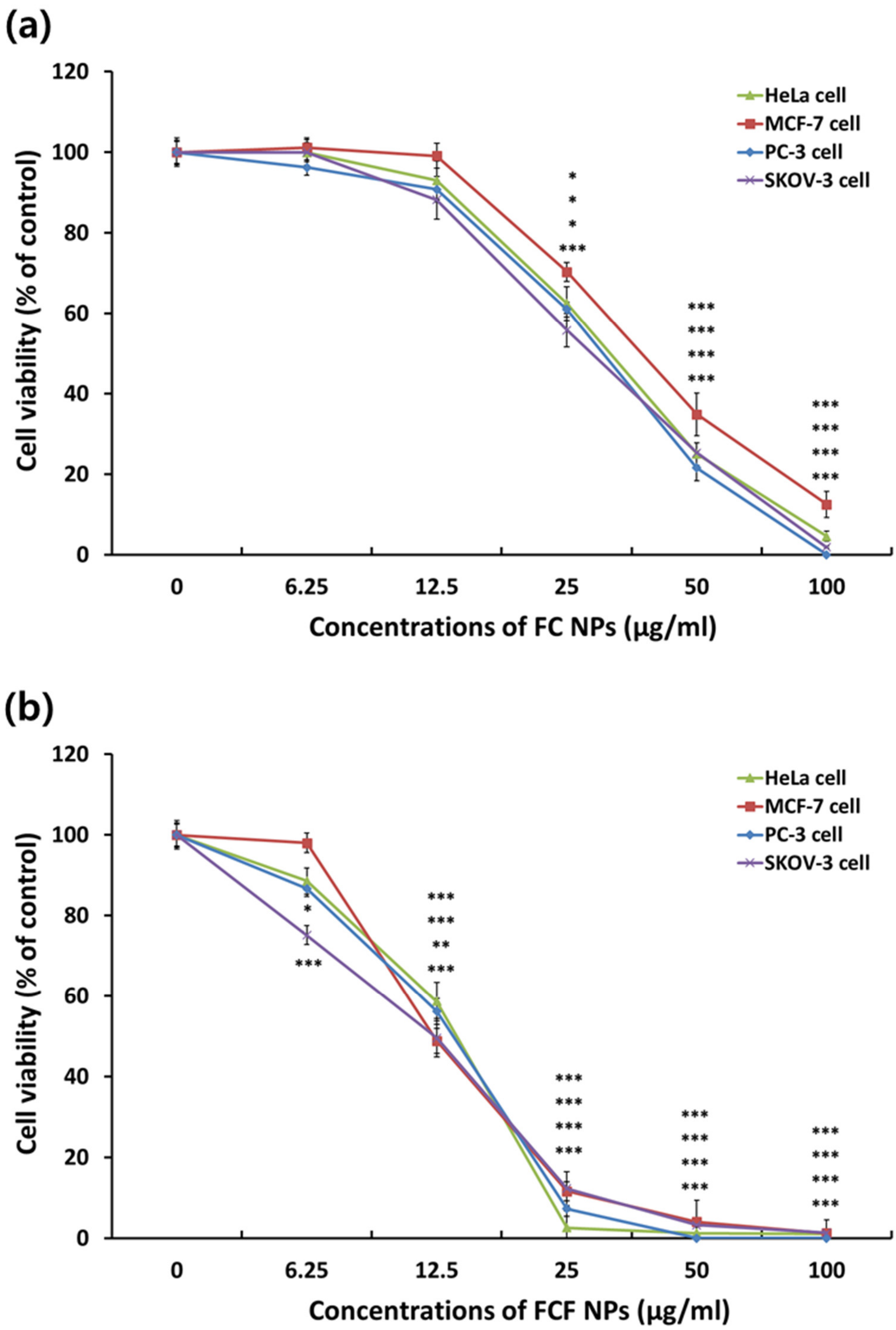

Figure S4. Photodynamic FC and FCF NPs anticancer activity in various cancer cells. Phototoxicities of the (a) FC and (b) FCF NPs in HeLa, MCF-7, PC-3, and SKOV-3 cell over 10-min exposure to 40 mW LED light. Data for photodynamic anticancer activity are expressed as mean ± S.D. (n = 4). Statistical significance was analyzed by Student's *t*-tests. *$p < 0.05$, **$p < 0.005$, ***$p < 0.0005$ (vs. control).

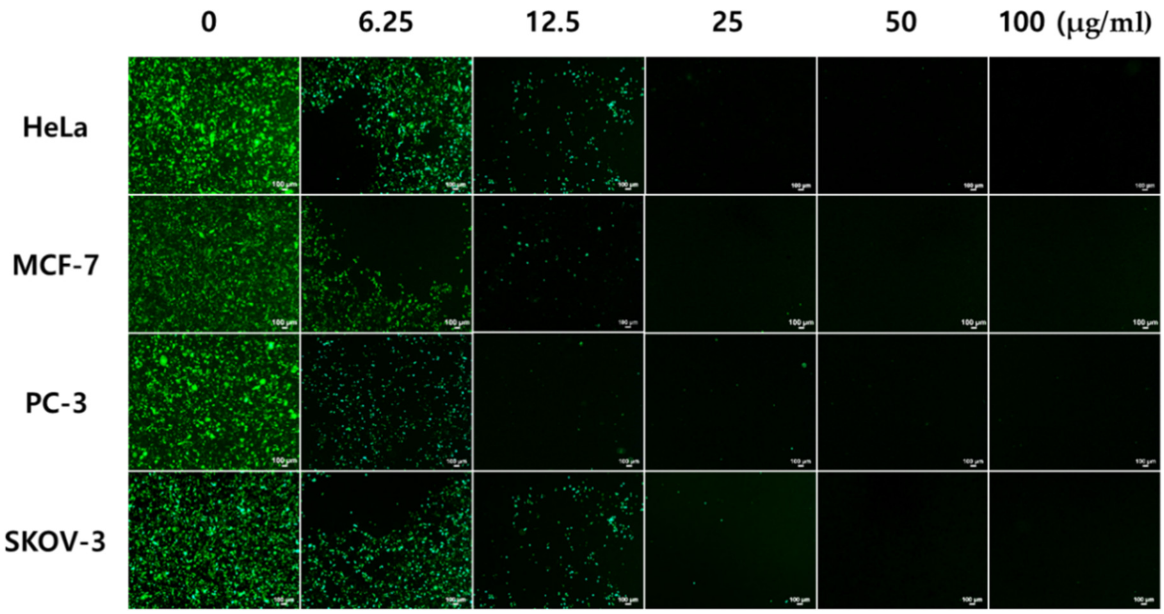

Figure S5. Fluorescence images showing phototoxicity of the FCF NPs in cancer cells. Fluorescence images of live cancer cells were taken 24 h after PDT (40mW) for 10 min. The images were acquired using a 10x objective lens and fluorescence optics (excitation/emission at 377/447 nm). Scale bar = 100 μm.
